# A new cross-platform architecture for epi-info software suite

**DOI:** 10.1186/s12859-018-2334-8

**Published:** 2018-10-22

**Authors:** Blake Camp, Jaya Krishna Mandivarapu, Nagashayan Ramamurthy, James Wingo, Anu G. Bourgeois, Xiaojun Cao, Rajshekhar Sunderraman

**Affiliations:** Department of Computer Science, Georgia State University, 25 Park Place, Atlanta, GA USA

**Keywords:** Cross-platform, Form-design, Analytics, Pubic-health, NoSQL, Electron, Data-collection

## Abstract

**Background:**

The Epi-Info software suite, built and maintained by the Centers for Disease Control and Prevention (CDC), is widely used by epidemiologists and public health researchers to collect and analyze public health data, especially in the event of outbreaks such as Ebola and Zika. As it exists today, Epi-Info Desktop runs only on the Windows platform, and the larger Epi-Info Suite of products consists of separate codebases for several different devices and use-cases. Software portability has become increasingly important over the past few years as it offers a number of obvious benefits. These include reduced development time, reduced cost, and simplified system architecture. Thus, there is a blatant need for continued research. Specifically, it is critical to fully understand any underlying negative performance issues which arise from platform-agnostic systems. Such understanding should allow for improved design, and thus result in substantial mitigation of reduced performance. In this paper, we present a viable cross-platform architecture for Epi-Info which solves many of these problems.

**Results:**

We have successfully generated executables for Linux, Mac, and Windows from a single code-base, and we have shown that performance need not be completely sacrificed when building a cross-platform application. This has been accomplished by using Electron as a wrapper for an AngularJS app, a Python analytics module, and a local, browser-based NoSQL database.

**Conclusions:**

Promising results warrant future research. Specifically, the design allows for cross-platform form-design, data-collection, offline/online modes, scalable storage, automatic local-to-remote data sync, and fast analytics which rival more traditional approaches.

## Background

Developed by the Centers for Disease Control and Prevention (CDC) [1], Epi-Info is a software package which enables public health workers to assess disease outbreak, collect data, manage surveillance data sets, and analyze data [2]. The Epi-Info software is widely used by the epidemiologists and health professionals in the governments, public health non-profits, NGO’s, universities and health schools (for example, [3–5]). It is estimated that there are over one million users [2]. The desktop software has undergone a number of revisions and is currently built upon the Windows operating system. While popular, it has become clear that there are several areas in which the product needs to be improved [6]. First, a particular deficiency of the system that needs to be addressed is the software’s inability to run on Linux or Macs. A tool that is truly capable of contributing to the international community’s fight against infectious diseases should support as many operating systems and devices as possible. An open-source and cross-platform version of such software package will allow the developers from around the world to access, design and enhance Epi-Info. Second, having been under development for more than three decades, Epi-Info is now comprised of several separate applications, codebases and use-cases including desktop, mobile, web, and cloud. This has resulted in an unfortunate increase in development complexity. Outbreaks can often spread faster than engineers can keep up. It is not uncommon for new analytics components or data-collection tools to be requested by public health teams on the ground during highly active outbreak situations. This on-the-fly requirements specification and engineering can be difficult to manage together with complicated codebases. Third, the existing interfaces for offline data-collection and maintenance protocols are not altogether intuitive. The processes for importing or broadcasting between remote servers and local client machines may present steep learning curves for public health officials.

In this work, we propose and implement a new cross-platform architecture for Epi-Info software suite, which can simplify the codebases, expedite the development process and incorporate open-source techniques for flexible interfaces. The proposed architecture adopts the Electron [7] as the cross-platform framework to achieve significant reduction in development time and cost. The open-source techniques in NoSQL and Python are also introduced into Epi-Info. NoSQL, as a viable database option, can scale extremely well and provide a flexible structure to otherwise unstructured data. Python [8] has emerged as a very popular languages for data analytics and becomes the coding language of choice for many in the science community. Its robust statistical libraries and machine learning frameworks make it a suitable choice for Epi-Info. In addition, the ease-of-use and platform universality from Python can greatly reduce the development time of any new modules in the event of some emergencies or outbreak.

A Web-Based Form-Designer is not currently part of the existing Epi-Info suite, but such a product has been needed for some time [2]. One challenge here is finding a balance between flexibility, speed, and ease-of-use with respect to the form design process. We propose to use AngularJS [9]. Even though AngularJS does not natively support drag & drop functionality, we develop a back-to-front design methodology to ensure a user-friendly, yet effective form-designer.

## Implementation

**Fig. 1:**
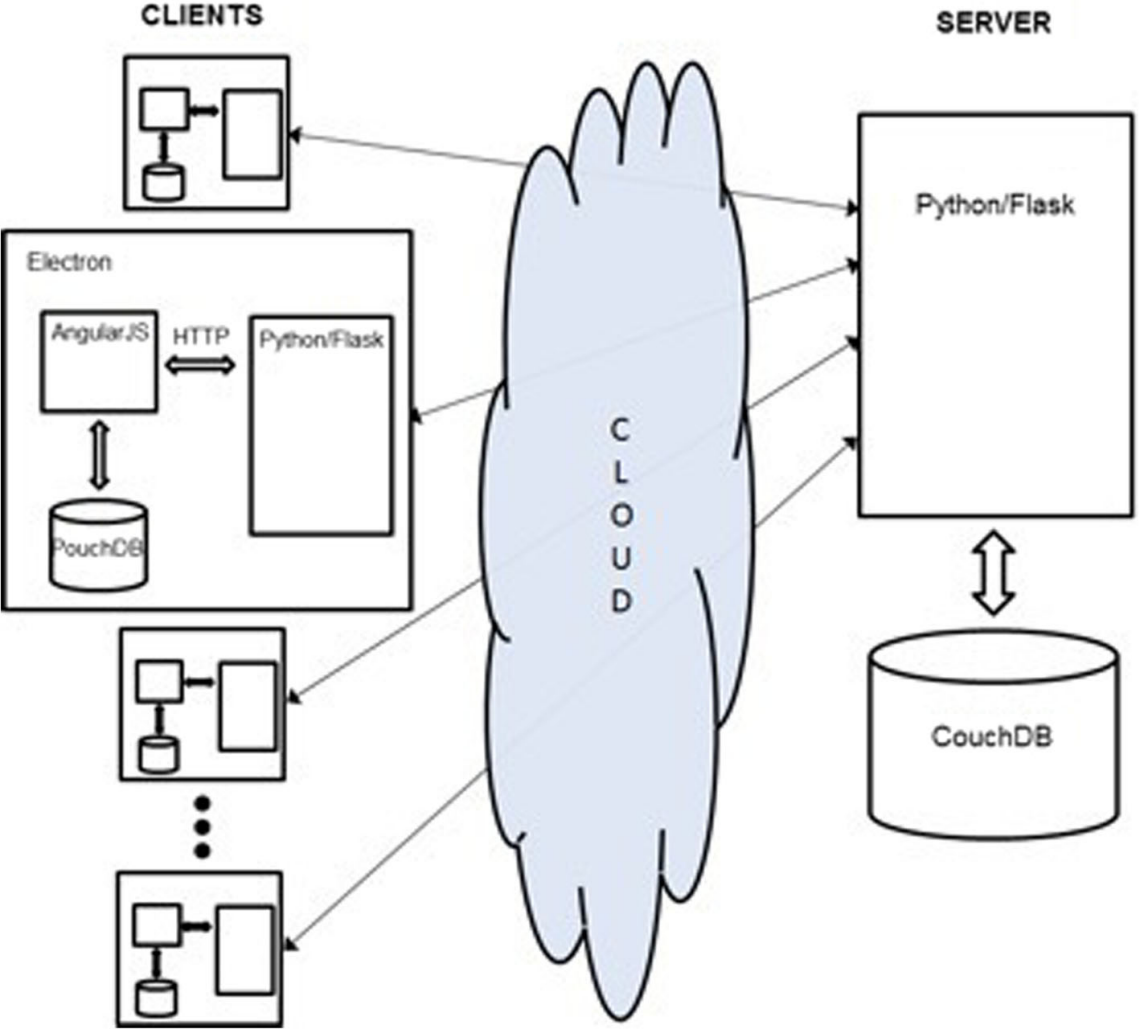
System Architecture - Local PouchDB clients sync automatically with a central CouchDB cloud server,allowing for seamless online-offline transition

We present a cross-platform system architecture which allows for intuitive form-design, data-collection, online and offline modes, automatic local-remote data sync, fast analytics, and scalable storage. The overall system architecture is shown in Fig. 1.

The Epi-Info deployment consists of

- A server-side CouchDB database which stores shared form templates, data, and other user information such as dashboards etc. This data is exposed as RESTful Web Services to the clients, and
- Multiple clients, each equipped with an AngularJS application that provides all the functionality of Epi-Info including form design, deployment of forms, data collection, and user dashboards, and on demand analytics. Each client stores its data in PouchDB, which is automatically synchronized the CouchDB on the server. The client also includes a Python/Flask module that provides access to a large set of analytics functionality. All of the client is encapsulated with the Electron, a platform-independent application framework.

### Client Side Architecture

To address the cross-platform system requirement, we use Electron as a wrap- per for an AngularJS front-end, a Python Analytics module, and an embedded NoSQL database called PouchDB.

**Fig. 2:**
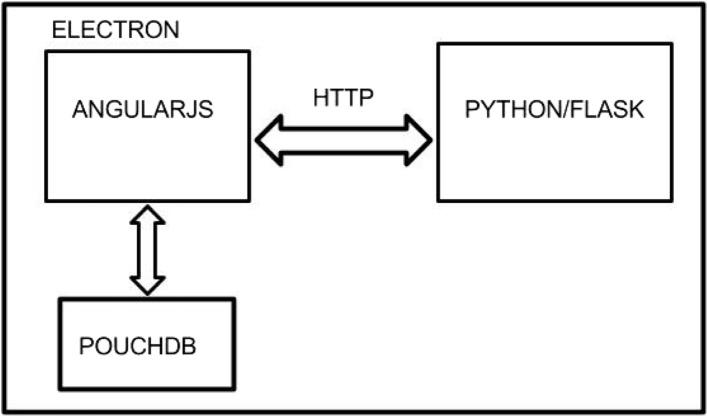
Client Side Architecture - An AngularJS app, Python Analytics Module, and local PouchDB

The database accessibility protocol was an important design consideration. PouchDB is a lightweight, browser-based NoSQL database which is designed to automatically sync with a remote Couch (Fig. 2) Database. However, the proper access point was not immediately obviously. As shown in Fig. 1, the PouchDB is accessed directly by the Angular front-end. Importantly, this configuration was chosen because the alternative approach, whereby the database is accessed directly by the Python Analytics module, would have required the use of a Python-PouchDB wrapper. The documentation for the wrapper is very light, and it has been much easier to use the original API’s for database interaction. Any data needed by the Python Analytics module can be requested and sent via a simple HTTP connection.

The Flask framework manages the Python code. When the Electron application is initiated, a child process is spawned which starts the Flask server, allowing access to the Python analytics module. This has proved successful and it has allowed us to seamlessly integrate Angular and Python in a single, local application. When an analytics requ-est is made, for example, the data is simply re-routed to the appropriate Python function via the HTTP connection.

Python was chosen because of it’s popularity and platform-agnosticism. It is critical that researchers from around the world be allowed to contribute to this project in a timely way. This can be facilitated by offering a platform comprised of tools which are popular and universal.

### NoSQL and PouchDB

NoSQL databases have been one of our primary areas of research to date. They are understood to scale extremely well because they are well-suited to provide a flexible structure to otherwise unstructured data. That fact has proven helpful when storing Epi-Form schemas. However, we have identified several other database-related issues which require careful consideration.

It was necessary to choose an appropriate candidate to be embedded with our Electron Application. This is critical because larger NoSQL databases, like MongoDB, require different installation protocols for different operating systems. Recall once again, our primary objective is to be a platform-agnostic application that is extremely user-friendly, and very little effort to download and install. Thus, our approach has been to embed a lightweight NoSQL database within our Electron desktop application.

After research, PouchDB was selected as the NoSQL database, and we consider it to be a viable option going forward. It’s robust documentation, community support, and seamless synchronization with CouchDB makes it very attractive. Furthermore, it is easy to embed, and can be interfaced directly with the Angular frontend. Specifically, PouchDB is designed to sync automatically with a remote Couch Database. This allows for seamless transition between online and offline modes, and guards against the potential for data-loss during transfer.

As a result of this auto-sync, any underlying changes to data on local client machines can be automatically broadcast a centralized remote database, and subsequently on to any additional client machines. Furthermore, PouchDB provides a detailed change-log which identifies and explains any alterations in local data or data-structure.

### Analytics Module

Epi-Info is essentially data-collection and analytics software. Consequently, the analytics module is perhaps the most crucial component, and the primary objective was to increase speed and efficiency. In the following paragraphs, we outline an approach which successfully mitigates the negative effects often found in cross-platform and NoSQL systems.

**Fig. 3:**
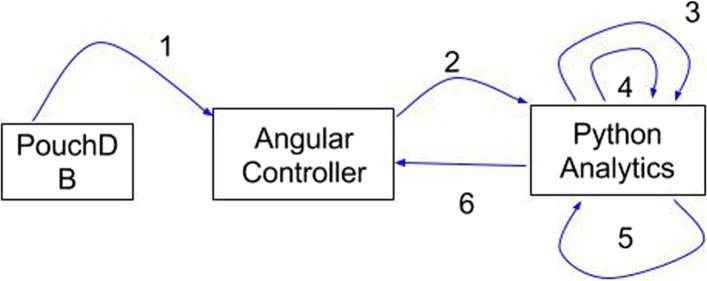
System Data-Flow - Each step requires time, see Table 1

It was important to precisely identify each point of data-transfer and manipulation in order to pinpoint any potential bottlenecks (Fig. 3, Table 1).

**Table 1:** Approximate time-requirements for critical data-flow processes

	Process	Time (Data: 50k x 200)
		(300MB)
1	Retrieve data from PouchDB	1 minute
2	HTTP POST, send JSON data to Python	30 seconds
3	Convert JSON to Pandas DataFrame	30–45 seconds
4	Compress/Write JSON to HDF	<1 second (compresses
		to only 21MB)
5	Analytics	varies, but fast
6	HTTP POST, return results to Angular	varies

Test Data: 50k records, by 200 features

In order to expedite the analytics cycle, we demonstrate drastic performance improvement by storing two copies of any particular dataset. We keep one copy in PouchDB, so that it may be available for automatic syncing with the central CouchDB. We keep another in a compressed format native to Python, called HDF5. Even on a slow machine, the read and write times for HDF5 are extremely fast, better even than SQL or CSV. Additionally, the excellent compression ratio means that even though we store the data twice, we increase the total storage-size requirement by less than 10%.

As shown in Table 1, the most costly processes, with respect to time, involve retrieving the data from PouchDB, sending the data to the analytics module, and converting the data to a useable DataFrame. This problem is exacerbated if the database is allowed to accumulate alot of data prior to carrying out these steps. Thus, it is possible to mitigate such effects by performing the operations iteratively, whenever new data is entered into the database. The compressed HDF5 DataFrame must be continuously maintained, allowing for immediate analytics requests at all times. Fortunately, PouchDB comes equipped witha change-log which offers a detailed explanation of any changes to the underlying data. This can be used to subsequently update the compressed DataFrame. The result is a system that would allow for very fast access to data and analytics which rivals even traditional approaches. Additional performance metrics are provided in the “Results” section of this paper.

### Form Designer

**Fig. 4:**
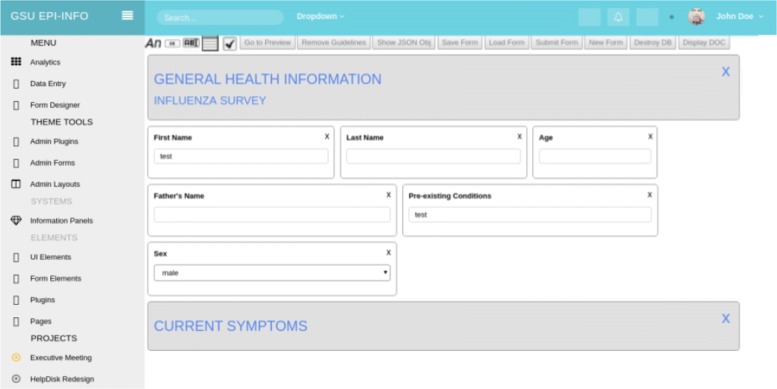
Screenshot of Angular-based drag-and-drop Form Designer

The challenge associated with building a web-based form designer is derived from a need to balance flexibility with specificity. The current desktop form-designer provided by Epi-Info offers extreme precision, allowing form-creators the ability to define form elements on a pixel-by-pixel basis. The form-schemas are then stored as XML, and the exact positions of form elements are subsequently recorded. On the one hand, this is desirable because health form appearance often requires such acute attention to detail. On the other, this can cause a large increase in design time. With our web-based AngularJS form designer, we strike a balance between the two characteristics, offering users an acceptable level of precision while simultaneously expediting the form-design process with a flexible and intuitive interface (Fig. 4).

## Results

To date, we have successfully generated executables for Windows, Mac, and Linux machines from a single code base, and we have shown that performance need not be completely sacrificed when building a cross-platform application.

**Fig. 5:**
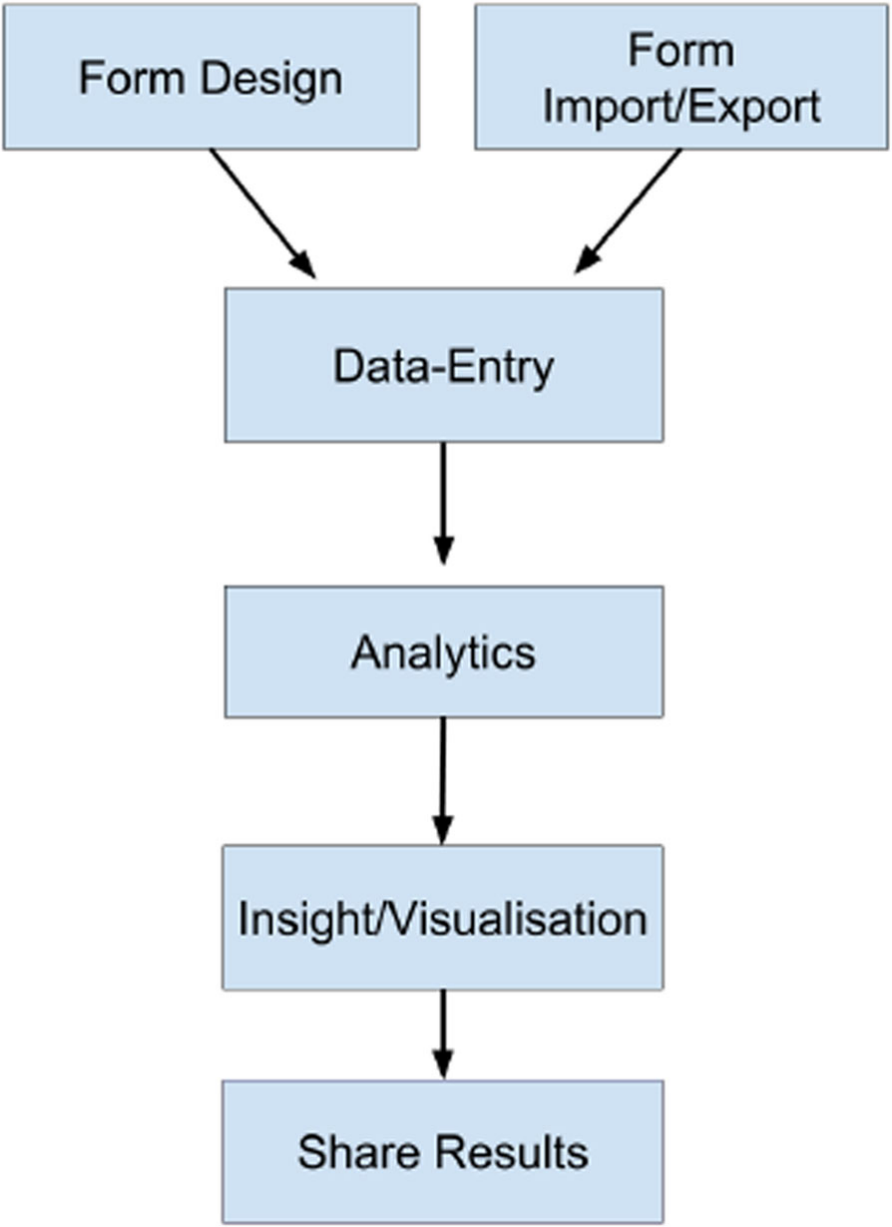
Typical Epi-Info workflow

**Fig. 6:**
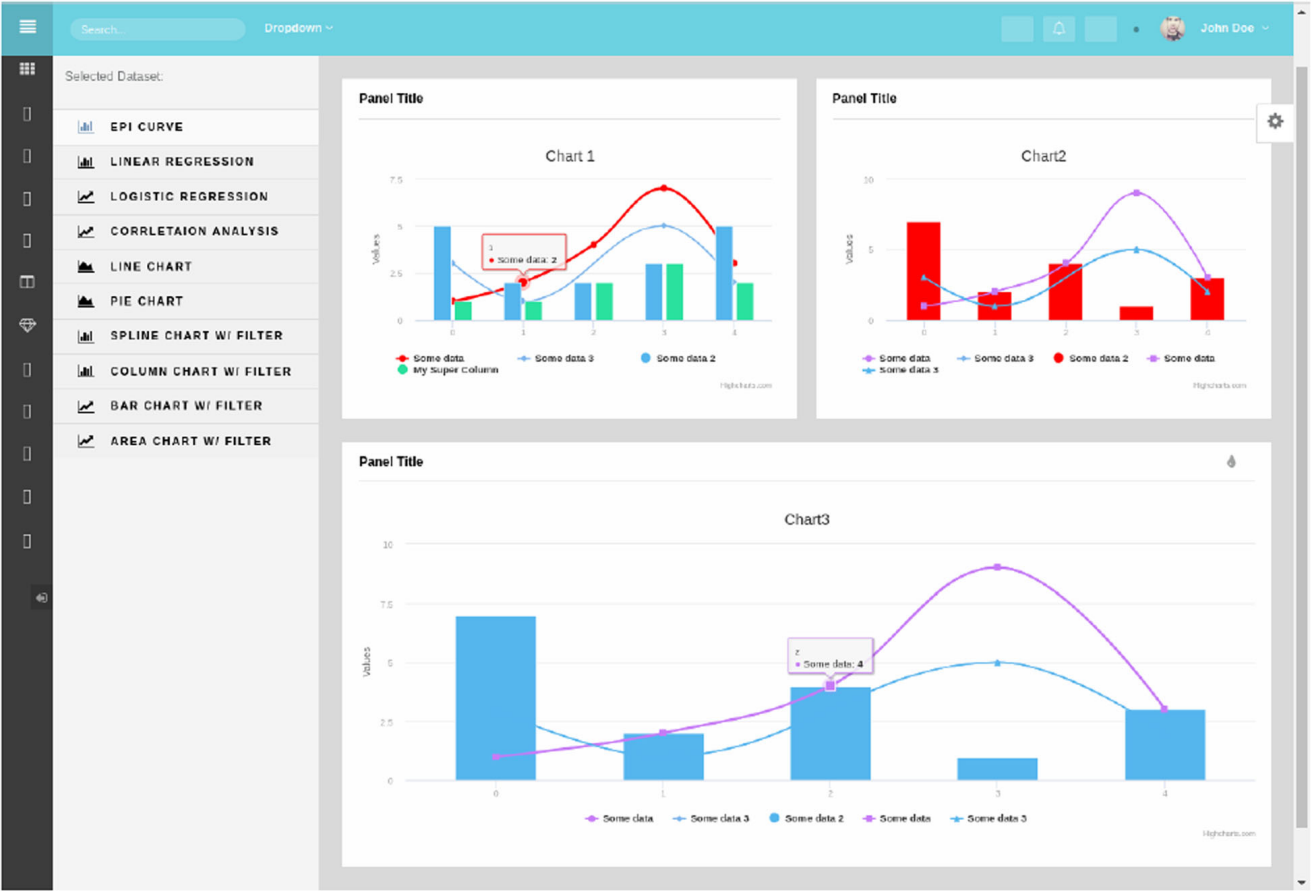
Screenshot of GSU Epi-Info Analytics interface

Figure 5 shows a typical Epi-Info desktop workflow. Our product can successfully support: Form Design, Import/Export of Forms to a centralized database, Data-Entry, Advanced Analytics, Savable and Customizable Advanced Analytics Dashboards (Fig. 6), and Report Export.

By incorporating the use of a compressed HDF5 DataFrame, we have successfully demonstrated that we can expedite the analytics cycle, thus mitigating many of the negative effects typically associated with cross-platform or NoSQL applications. For a dataset with 50,000 records and 200 columns, the software can read the data, perform a user- defined 10-variable multiple logistic regression, and report the results in under 2 s, even on modest machines.

**Fig. 7:**
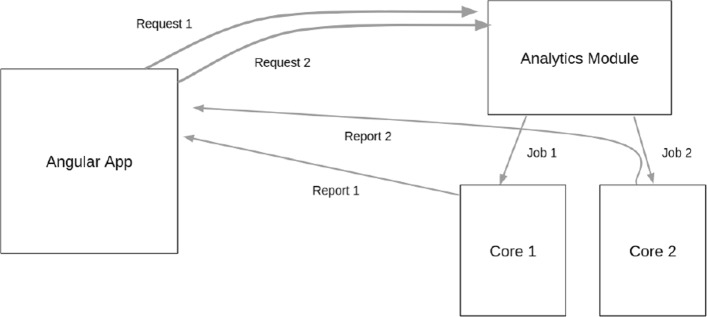
High level view of step-by-step analytics optimization using multiple cores

Additionally, the use of multiple cores can further optimize the analytics module. This allows multiple analytics requests to be made on-the-fly as needed. Reports are sent back to the user-interface as those jobs are completed. That is, any single request need not wait for a previous job to finish as long as there is another core available for use on the machine (Fig. 7).

## Additional Considerations

The design presented in this paper should be regarded as one acceptable approach with respect to the the aforementioned requirements. However, numerous other frameworks, architectures, and configurations could potentially prove adequate. In this section, our reasons for favoring this system will be explained more thoroughly.

The research conducted during the course of this project resulted in discussions with several additional research groups. Of particular note, was a collaborative multi-day meeting with a team from the University of Brasilia and representatives from the Itaipu Bi-National Energy Plant. During the meeting, a consensus was articulated which highlighted the need for greater amounts of international standardization and collaboration as separate nations and organizations seek to fight the spread of infectious diseases, particularly with respect to the technology involved. On that front, there was additional agreement that there are two domains where this is particularly important: data standardization, and software standardization.

The standardization of data is a challenging task, but progress has been made thanks in part to Health Level Seven International (HL7). Recently they have published a standard for public-health data knows as the FHIR, and it is currently being incorporated into various software tools around the planet. There seems to be less cohesion, however, on the software standardization front. This can be attributed to the enormous amounts of specific use-cases, location-specific needs, and a disjoint international community of engineers. Indeed, the CDC often plays a leadership role in many areas of the world in the event of outbreaks. Nevertheless, there are countless other organizations, such as Itaipu, which each have separate teams building unique tools to combat specific regional problems. Consequently, it appears there is a fair amount of redundancy with respect to functionality and code. This problem is likely to persist without the oversight of an international standardizing orginization. However, it is possible that the problem could be mitigated, even slightly, by the use of broadly-adopted and flexible technologies. When appropriate, generality and popularity should be favored.

Central to the initial conceptualization of our design was the selection as Python as the language of choice for the Analytics Module. As mentioned, this was due in large part to its popularity among the scientific computing community and its platform agnostic quality. This ultimately factored greatly into the choice of a suitable cross-platform framework. Framework candidates which were discussed included Electron, Kivy, and.NET-Core. Electron and Kivy were selected for closer inspection due to language familiarity amongst the design team.

Kivy is a cross-platform framework for developing Python apps. It runs on iOS, Linux, Windows, Android, and OSX; making it very attractive. However, it would have required time to become acquainted with the front-end framework provided by Kivy, as it does not rely on traditional web-technologies. Ultimately, this encouraged us to move towards Electron.

Electron, as opposed to Kivy, allows engineers to work with familiar technologies which can be easily encapsulated in the framework. We feel that this should allow for increased flexibility, a reduction in development time, and greater ability to share components across applications. It is not clear, for example, that it would be easy to deploy the majority of a Kivy app to the web. However, the Electron app we have designed should be fairly easy to migrate. Additionally, there are mobile frameworks, such as Ionic, which would also facilitate the simple transfer of the majority of code to a mobile app.

We were greatly encouraged by the technological similarity presented by the group from Itaipu. Like us, they use a combination of AngualarJS and Python. Their current app, however, is entirely web-based, yet they have a need for offline capability. Because we both are using highly flexible, similar technologies, there is a real opportunity for collaboration and outright code-sharing. We feel it would be easy to extend their application, wrap it in an electron framework with an embedded NoSQL database, and allow for a robust offline use-case. This would simply not be possible if each group were not using such widely adopted technologies, and it shows the power and need for additional software standardization.

Importantly, new technologies must first be examined and evaluated based on the quality of community support. Public-Health is a critical domain, and it would not be wise to imprudently experiment with untested and weakly-supported tools. This was discovered first hand by our researchers, particularly when tasked with choosing an acceptable local NoSQL database. Many newer, lightweight NoSQL databases have very shallow documentation and community support. ForerunnerDB was one such product which ultimately proved impractical. Development was drastically slowed whenever a small bug was found in the database code because the required documentation literally did not exist. Eventually, PouchDB was discovered, and we found it to have adequate support, greatly simplifying and expediting the development process.

## Conclusions

Creative system design can alleviate many of the undesirable qualities typically associated with cross-platform frameworks, such as Electron. This can require a mix of languages, databases, and design patterns. The power of the resulting system has, in this case, proven to be worth the effort, successfully addressing many of the necessary system requirements.

With our cross-platform framework design for Epi-Info, the international community will now have the tools to rapidly respond to an emergency outbreak, even under remote conditions. By designing a single code-base that is capable of generating executables for multiple platforms, developers can quickly provide customized components to those deployed. Our work towards optimizing the data analytics will enable better coordination and a more effective response to any outbreak around the globe. However, future research is still needed, such as the development and deployment of Epi-Info on mobile devices, including hand held tablets and smart phones. It is also imperative to develop methods to automate and adapt data synchronization with centralized servers/coud in an opportunistic way when connectivity is available.

## Availability and Requirements

**Project name:** GSU Epi-Info

**Project home page:**http://epi.cs.gsu.edu & https://bitbucket.org/bbc1183/epi-info/

**Operating system(s):** Platform independent. However, there are known issues with respect to installing electron on Ubuntu 16.04. Recommend Ubuntu 14 for development.

**Programming language(s):** NodeJS, AngularJS, Python

**Other requirements:** In the repository located at https://bitbucket.org/bbc1183/epi-info/, the active branch is called ’dev’. The current software is located in the directory called ’electron-with-python’. There is a readme.txt in the root of that directory. Will not work out of the box on Ubuntu 16.04.

**License:** MIT

**Any restrictions to use by non-acedemics:** Not applicable.

## Abbreviations

CDC: Centers for Disease Control and Prevention
HDF5: Hierarchical Data Format (HDF) is a set of file formats (HDF4, HDF5) designed to store and organize large amounts of data
NoSQL: Non-relational database
